# Observations on Salivary Calculus Found in the Duct of Wharton on the Right Side

**Published:** 1857-07

**Authors:** E. Demorey


### ARTICLE VI.

*Observations on Salivary Calculus found in the Duct of Wharton on the right side.* By DR. E. DEMOREY. Translated from the French of L'Art Dentaire, for the Am. Jour. Dent. Science.

B..., (Jean Baptiste,) mason, aged thirty-two years, of a very good constitution, came in September, 1855, to consult my father, (I being present,) for a swelling under the tongue.

*Antecedents.*—The patient stated that at the age of twelve years he discharged a small stone, of the size of a grain of barley, which he recognized by the hardness of its texture as a stony concretion.

While eating, this concretion gave rise to a soft painful tumor of the size of a hickory nut, situated on the right side of the frenum under the tongue. The patient was often compelled to stop eating with a view of freeing his mouth from liquid before he could continue his meal; it was particularly while eating spiced meats, exciting a large salivary secretion, that he perceived the tumor was rapidly forming. No very decided inflammatory symptoms were manifested during its growth.

I was not able to ascertain from the patient at what period the beginning of this affection commenced.

It was while eating that this first stone escaped; it had upon its surface a small quantity of bloody puss; the salivary tumor was not immediately reproduced after the removal of the concretion, although there was a painful tension in the region of the submaxillary gland. The movement of the tongue and mastication was more free. Notwithstanding this sensible amelioration there was always a little uneasiness produced by a small induration which did not increase much; at a later period the movements of the tongue became more difficult and speech embarrassed.

At the end of five or six months the patient states that he discovered on the right side of the frenum, under the tongue, a small hard tumor of the size of a Lima bean, which was nothing but the development of the induration which had remained stationary for fifteen or twenty months. From this time the growth became manifest, the salivary secretion became more abundant, thicker than usual, a slight elastic swelling showed itself while eating, but often disappeared at the end of his meal; at the same time sharp pains extended to the corresponding parotidian region with tumefaction perceivable from the salivary gland.

In the month of April, 1855, the patient perceived that the mouth of the duct of Wharton was considerably dilated, (which was the cause of sharp pains,) and calculus was there seen. The swelling and pain slowly increased, the saliva became purulent; the calculus became more and more prominent and struck against the teeth when the patient moved his tongue.

*State of the Patient.*—When he presented himself, on the 9th of September, 1855, the pains were more acute; he was scarcely able to open his mouth. Upon examining the interior of this cavity, the tongue being raised up, we found upon the floor of it, under the anterior part of the tongue on the right side of the frenum, and behind the orifice of the duct of Wharton, a tumor obliquely placed, of the size of an almond, elongated, cylindrical, prominent, and excessively painful, having little mobility and hard to the touch; when taken between the fingers, a foreign body was distinctly felt which offered resistance; an enormous calculus was discovered to be contained in a small quantity of liquid, seeking egress from the duct of Wharton already largely dilated. The anterior extremity of the concretion, which had extended out a considerable distance, was smooth, polished, and of a dirty yellow color; the submaxillary gland was double the size of that of the opposite side; it was after the extraction of the calculus that this difference was more manifest.

We extracted the calculus by seizing it with a pair of forceps. After the operation, which was effected without any resistance, a quantity of thick, purulent salivary liquid escaped; the painful symptoms immediately disappeared.

We convinced ourselves by sounding, that there were no more concretions present; the duct was largely dilated.

The next day the orifice had considerably contracted and the duct was very much reduced. The cure was completed in a few days.

I regretted extremely not having examined the patient this year, and assuring myself of his perfect restoration.

The continuance of the uneasiness, the presence of the small induration after the ejection of the first concretion, induced me to think that a relapse would certainly take place, there being two calculi in the duct of Wharton. The largest was the first thrown out; the second continued to develop but very slowly.

In fact, it was only necessary to examine the calculus, to be convinced, from its size, that it must have taken a long time to form.

*Physical Aspect of the Calculus.*—The calculus which came under our observation, was very large and regularly developed; was elongated, rounded, thicker in its center than at its extremities, it resembled a spindle slightly turned in the form of an S, it was flattened a little upon its lateral sides, its faces not exactly equal, presenting in some points slight depressions. It was hard and compact; its color, of a yellowish white; and its surface was covered with small papillæ, which gave it a fretted appearance. Its two extremities were rounded; these small grains, which were upon the one that presented itself in the interior of the mouth, had disappeared. It was very smooth, and seemed to have been polished and worn by the soft parts with which it had come in contact.

Examined with a high power, its surface was covered with small papillæ, varying somewhat in size and form, and grouped together in such manner as to form tortuous furrows, which performed imperfectly the office of drains, such as have marked other calculi.

We observed, moreover, the presence of irregularly discriminated capillary orifices. A section made from this calculus, enabled us to see plainly two substances; the central one white, large, and which constituted the nucleus; the other, on the outside, thicker, yellowish, of the same texture as the first, forming a concentric layer, very visible, and made up of many superimposed layers. It could not be seen with the naked eye as far as the nucleus; the line of demarcation extend between those exterior layers and the surface, and this was only to be seen by the aid of the microscope; thus the calculus was formed of three substances. Its weight, obtained by the aid of an excellent balance, was 2.4915; its density, 1.9025. In size it was 42 millemetres, and its greatest circumference 28 millimetres.

*Microscopic Examination.*—A portion of the matter submitted to microscopic examination, shows us, amongst some badly defined crystals of calcareous substances, fragments of the lomellæs of prominent epithelium, of which the nuclei were more or less deformed and smoothed by compression. These epithelial lomellæ were analogous to those found in the saliva. This is further proof that the salivary liquid is the source of submaxillary calculi.

In conclusion, we give the analysis made by my friend M. Humbert:

| | |
|---|---|
| Phosphate of lime, . . . . . . | 66.70 |
| Carbonate of lime, . . . . . . . . | 11.30 |
| Animal matter, . . . . . . . | 20.00 |
| Magnesia, oxyd of iron, chloride of sodium, sulphates, sulpho-cyanuret of sodium, . . | 2.00 |
| | 100.00 |

*Chemical Analysis.*—Pulverized and washed with distilled water, the calculus yielded chloride of sodium, a small quantity of sulphate of lime, and appreciable traces of a sulpho-cyanuret alkali, and more of animal matter, which gave to the solution when heated, a decided salivary odor, which was particularly manifest when the saliva was brought in contact with a very warm metallic substance. The reaction was alkaline. A weak solution of potassa threw down from the substance a strong proportion of organic matter, of an albuminous nature. The presence of albumin was undoubted.

Such is the rare and interesting case which accidently came under my observation.—*Gazette des Hôpitaux.*

